# Spatial omics of neuroinflammation: insights across brain diseases

**DOI:** 10.3389/fimmu.2026.1923995

**Published:** 2026-09-07

**Authors:** Mingming Li, Qianying Wang, Jian Li, Yanjun Liu, Lixia Chen, Pei Liu, Jia Guo

**Affiliations:** 1 Department of Neurology, Lanzhou University Second Hospital, Lanzhou University, Lanzhou, China; 2 Gansu Provincial Neurology Clinical Medical Research Center, Lanzhou University Second Hospital, Lanzhou, China; 3 Expert Workstation of Academician Wang Longde, Lanzhou University Second Hospital, Lanzhou, China; 4 Department of General Surgery, Lanzhou University Second Hospital, Lanzhou University, Lanzhou, China

**Keywords:** brain diseases, microglia, inflammatory niches, neuroinflammation, spatial omics, spatial transcriptomics

## Abstract

Neuroinflammation is a common pathological feature of diverse brain diseases, but inflammatory activity is rarely distributed uniformly across diseased tissue. Instead, microglia, astrocytes, infiltrating immune cells and inflammatory mediators are often organized around specific pathological structures, including amyloid plaques, demyelinated lesion rims, ischemic borders, necrotic tumor regions and perivascular white matter compartments. Although bulk and dissociation-based single-cell approaches have defined many inflammatory cell states, they cannot determine where these states reside, how they relate to local pathology, or whether inferred cell–cell interactions occur within plausible spatial neighborhoods. Spatial omics addresses this limitation by preserving molecular information within intact tissue architecture. In this review, we summarize major spatial transcriptomic, proteomic, metabolomic and same-section multi-omic technologies, with emphasis on the types of neuroinflammatory questions each platform can answer. We then examine how spatial omics has reshaped the understanding of neuroinflammation across Alzheimer’s disease and tauopathies, multiple sclerosis, ischemic stroke, glioma, infection-related neuroinflammation and aging. Across these settings, spatial studies have revealed plaque-associated glial niches, lipid- and iron-enriched lesion rims, core–penumbra inflammatory zonation, and hypoxic or perivascular immune microenvironments. These findings suggest that neuroinflammation should be understood not only as a set of molecular or cellular states, but also as a spatially organized tissue process shaped by local pathology, cellular adjacency and microenvironmental gradients and spatial compartments. Finally, we discuss the limitations of current spatial maps, including resolution, human tissue constraints and insufficient functional validation, and outline future directions toward integrated, temporal and clinically translatable spatial atlases.

## 1 Introduction

Neuroinflammation is a fundamental component of many central nervous system (CNS) diseases, including Alzheimer’s disease, multiple sclerosis, ischemic stroke, glioma, infection-associated injury and aging-related white matter degeneration (1). It involves coordinated responses of microglia, astrocytes, endothelial cells, infiltrating immune cells and neuronal populations, and is often accompanied by cytokine production, complement activation, oxidative stress, metabolic remodeling and synaptic or myelin injury (2). However, neuroinflammation is not simply a global increase in inflammatory mediators. In diseased brain tissue, inflammatory activity is frequently concentrated around specific pathological structures, such as amyloid plaques, demyelinated lesion rims, infarct borders, necrotic tumor regions and perivascular spaces. This spatial organization cannot be fully understood by measuring cell types or gene expression alone.

Bulk assays obscure regional heterogeneity, whereas dissociation-based single-cell approaches recover cellular diversity at the cost of anatomical context. In neuroinflammation, this limits the assignment of disease-associated cell states and candidate interactions to specific lesions, tissue compartments and cellular neighborhoods (3). Spatial omics technologies directly address this gap by preserving molecular information within intact tissue architecture. Spatial transcriptomics was developed to map gene expression back to histological position, allowing tissue regions and molecular states to be analyzed in their native context (4). Subsequent approaches have expanded this principle through higher-resolution sequencing, imaging-based transcript detection, spatial proteomics and same-section multi-omics, enabling researchers to link RNA, protein, pathology and cellular neighborhoods within a shared coordinate system (5–7). For neuroinflammation, this is particularly valuable because inflammatory niches are defined by local adjacency, gradients and tissue-specific microenvironments rather than by isolated cell states.

Recent spatial studies have begun to redefine neuroinflammation across major brain diseases. In Alzheimer’s disease, spatial transcriptomics identified plaque-associated molecular programs and multicellular inflammatory responses organized around amyloid deposits (8). In multiple sclerosis, MRI-informed single-nucleus profiling and spatial validation revealed a lymphocyte–microglia–astrocyte axis at the chronically inflamed lesion edge, linking local immune organization to smoldering demyelination (9). In ischemic stroke, integrated single-cell and spatial transcriptomics distinguished ischemic core-associated and penumbra-associated microglial states with different inflammatory and metabolic properties (10). In glioblastoma, integrated spatial transcriptomic and proteomic analyses have shown that malignant and immune states are arranged into multilayered niches shaped by hypoxia, necrosis and vascular proximity (11).

Together, these findings suggest that neuroinflammation is not only disease-specific but also niche-specific. Although the initiating insults differ, spatial studies repeatedly reveal a convergent architecture composed of a pathological focus, nearby myeloid activation, surrounding reactive glial states, graded metabolic or complement activity and locally constrained cell–cell communication. In this review, we summarize major spatial omics technologies, examine their application across representative brain diseases, extract emerging principles of spatially organized neuroinflammation, and discuss how spatial maps can be advanced from descriptive atlases toward mechanistic and clinically translatable models.

### 1.1 Search strategy and selection criteria

This structured narrative review was informed by searches of PubMed/MEDLINE and preprints from bioRxiv and medRxiv indexed in Europe PMC, covering the period from database inception through August 20, 2026. PubMed/MEDLINE was selected as the primary bibliographic database because this review focuses on biomedical and neuroscience applications and was designed as a structured narrative synthesis rather than an exhaustive systematic review; Scopus, Embase, and Web of Science were therefore not searched, and no claim of exhaustive coverage is made. The technology search block included “spatial transcriptomics,” “spatial multi-omics,” “spatial proteomics,” “spatial metabolomics,” “spatial epigenomics,” “spatial ATAC-seq,” “spatial CUT&Tag,” “mass spectrometry imaging,” “MALDI imaging,” “DESI imaging,” “*in situ* sequencing,” “imaging mass cytometry,” “multiplexed ion beam imaging,” and “MIBI-TOF,” together with relevant platform names. These terms were combined with neuroinflammatory terms, including “neuroinflammation,” “microglia,” “astrocyte,” and “myeloid,” and disease-context terms covering Alzheimer’s disease, neurodegeneration, multiple sclerosis, stroke, cerebral ischemia, glioma, glioblastoma, central nervous system infection, and brain aging. The electronic searches identified 1,640 records (1,338 from PubMed/MEDLINE, 290 from bioRxiv, and 12 from medRxiv). After duplicate records and preprints superseded by peer-reviewed publications were removed, 1,457 unique records remained for title and abstract screening. Following title and abstract screening and supplementary reference-list searching, 109 publications were assessed in full and retained in the final narrative synthesis. Only English-language publications were considered, and preprints were retained only when no peer-reviewed version was available.

## 2 Spatial omics technologies

### 2.1 Spatial transcriptomics: sequencing-based vs imaging-based

Two technological families dominate, with complementary strengths. Sequencing- or capture-based methods place a tissue section on a surface patterned with spatially barcoded probes that bind polyadenylated mRNA; after reverse transcription and sequencing, each transcript is mapped to the coordinate of its capture barcode. Introduced by Ståhl and colleagues (4) and commercialized as 10x Visium, the approach has since been refined by Slide-seq and Slide-seqV2 (12, 13), high-definition spatial transcriptomics (14), Stereo-seq (15) and DBiT-seq (7), progressively shrinking the capture unit from ~100 μm spots toward subcellular spacing. Their advantage is unbiased, transcriptome-wide measurement, which lets novel programs be discovered rather than merely re-detected; their constraint is resolution, as a capture unit on the order of tens of micrometres typically overlaps several cells whose transcripts must then be computationally deconvolved.

Imaging-based methods detect transcripts directly in tissue at single-cell to subcellular resolution. Many hybridization-based platforms, such as MERFISH and seqFISH+, use predefined gene panels, whereas *in situ* sequencing methods such as FISSEQ can support untargeted or transcriptome-scale profiling. Hybridization-based single-molecule methods - seqFISH and seqFISH^+^ and multiplexed error-robust FISH (MERFISH) - identify transcripts through combinatorial rounds of probe binding and imaging, scaling from dozens to thousands of genes (16, 17). *In situ* sequencing methods - the original *in situ* sequencing (ISS) (18), FISSEQ (19) and STARmap (20), the last extended as STARmap PLUS to co-read transcripts with amyloid and tau protein in one section - amplify and sequence barcodes directly within the tissue. Commercial single-cell platforms such as Xenium (21), MERSCOPE and CosMx have made these approaches widely accessible. By preserving each transcript’s exact position, imaging-based methods allow the radial and gradient organization of a niche to be measured directly.

The two families are complementary rather than ranked: capture-based methods are the instrument of discovery, imaging-based methods that of precise localization and validation, and the strongest studies pair them.

### 2.2 Spatial proteomics

Because glial inflammatory states are ultimately enacted by proteins, and transcript abundance is an imperfect proxy for protein level, antibody-based spatial proteomics is indispensable, particularly in human tissue. These methods quantify antibody-defined targets at single-cell or subcellular resolution while preserving tissue architecture, and fall into three families. Metal-tag, mass-based methods conjugate antibodies to rare-earth isotopes read out by mass spectrometry, avoiding the autofluorescence and spectral overlap that limit immunofluorescence: imaging mass cytometry (IMC) (6) ablates the tissue with a laser, whereas multiplexed ion beam imaging (MIBI, later MIBI-TOF) (22, 23) uses an ion beam, both resolving on the order of forty markers. Cyclic-fluorescence methods — tissue-based cyclic immunofluorescence (t-CyCIF) (24) and co-detection by indexing (CODEX, now PhenoCycler) (25) — build high-plex images through iterative staining, imaging and signal removal. Region-based digital methods, exemplified by GeoMx digital spatial profiling (26), count oligonucleotide barcodes released from user-defined regions and can profile high-plex protein or whole-transcriptome targets, trading native single-cell resolution for plex. Spatial proteomics measures protein abundance and localization, providing information closer to the effector level than RNA expression and resolving features inaccessible to transcriptomics. However, protein abundance alone does not demonstrate biological function. These platforms also operate at lower plex, require predefined and carefully validated antibodies, and do not support *de novo* target discovery.

### 2.3 Spatial metabolomics and multimodal spatial integration

Two further frontiers remain least developed in neuroinflammation yet are conceptually central, since the recurrent inflammatory program is itself metabolic and lipid-centred. Spatial metabolomics is driven mainly by mass spectrometry imaging (MSI), which samples a section pixel by pixel — by MALDI, DESI or secondary-ion approaches (27) - to map hundreds of lipids and metabolites label-free, with single-cell methods such as SpaceM (28) and SEAM (29) pushing toward cellular resolution. Because lipid-laden, cholesterol-handling myeloid states are defined by their lipid cargo, MSI can read that cargo directly rather than infer it from transcripts, and the lipid-rich myelin of the brain makes neurolipidomics especially informative; its principal constraints are coarser spatial resolution and the difficulty of co-registering metabolite images with individual cells. Spatial multi-omics should be distinguished from multimodal integration of separately acquired datasets. In many studies, transcriptomic, proteomic, metabolic or pathological measurements are generated independently, often from serial or adjacent sections, and subsequently aligned computationally; such approaches do not measure multiple molecular layers within the same tissue section. By contrast, same-section spatial multi-omics measures two or more molecular modalities within a shared tissue section. DBiT-seq can co-profile RNA and proteins, whereas STARmap PLUS and Visium protein co-detection map transcripts together with protein or pathological signals. Spatial-CUT&Tag and spatial-ATAC-seq are spatial epigenomic methods and, when used alone, should not be classified as multi-omic assays, although they can provide an epigenomic component within a multimodal experimental design (30, 31).

### 2.4 Which platform answers which inflammation question

No single platform meets every requirement, so the choice should follow from the question being asked. Discovering what inflammatory program surrounds a pathological focus, with no prior hypothesis about its constituents, calls for unbiased capture-based transcriptomics, whose whole-transcriptome coverage lets a novel program emerge *de novo* before deconvolution assigns it to cell types. Establishing which cell type occupies which position - its distance from a lesion, or how its state partitions across a lesion’s compartments - requires single-cell-resolution imaging transcriptomics, since only preserved single-transcript coordinates resolve radial and gradient architecture. Testing whether a mouse-defined state is real in the human brain, expressed as protein, and resident rather than infiltrating, is the province of antibody-based spatial proteomics. Reading the metabolic or lipid state of a niche *in situ* points to spatial metabolomics and immunometabolic imaging panels. Inferring how co-localized cells communicate demands single-cell resolution, because the proximity constraint that lends such inference credibility can be imposed only where cells are individually resolved. Resolving the integrated, multi-layer regulatory state of a niche requires same-section multi-omics, which remains nascent. Two considerations cut across every choice: whether the tissue is fresh-frozen or formalin-fixed, which constrains platform eligibility and weighs heavily for scarce human material, and whether the immediate goal is hypothesis generation or validation. Because most questions here demand both transcriptome-wide breadth and single-cell precision — which no current platform delivers at once - the most informative neuroinflammation studies are deliberately multi-platform (**Table 1**).

**Table 1:** Major spatial omics platforms for neuroinflammation research.

Platform category	Representative methods	Main readout	Key advantage	Main limitation	Best use in neuroinflammation
Capture-based spatial transcriptomics	Spatial transcriptomics/10x Visium, Slide-seq (4, 12)	Whole-transcriptome RNA expression with spatial coordinates	Unbiased discovery of spatial gene programs	Limited single-cell precision in some platforms; often requires deconvolution	Identifying inflammatory programs around plaques, lesion rims, infarct borders or perivascular niches
Imaging-based spatial transcriptomics	MERFISH, seqFISH+ (16, 17)	Targeted or untargeted *in situ* transcript detection	Single-cell or subcellular localization	Restricted gene panels in many platforms; image-segmentation artifacts	Mapping cellular neighborhoods, radial organization and local inflammatory gradients
Spatial proteomics	IMC, GeoMx DSP (6, 26)	Multiplexed protein expression in intact tissue	Direct protein-level validation of cell states	Lower plex than transcriptomics and antibody dependence	Validating reactive microglia, astrocytes, infiltrating myeloid cells and pathology-associated immune niches
Spatial metabolomics	SpaceM, SEAM (28, 29)	Lipids, metabolites and metabolic states	Direct measurement of metabolic and lipid remodeling	Coarser spatial resolution and difficult cell-type assignment	Studying myelin-derived lipids, cholesterol handling, oxidative stress and immunometabolism
Spatial epigenomics	Spatial-CUT&Tag, spatial ATAC-seq (30, 31)	Spatially resolved histone modifications or chromatin accessibility	Maps regulatory states while preserving tissue architecture	Single-modality when used alone; technically demanding	Defining epigenomic programs associated with inflammatory niches
Same-section spatial multi-omics	DBiT-seq, spatial- STARmap PLUS (7, 32)	Co-measured RNA and protein or pathological signals within the same tissue section	Links multiple molecular layers within a shared tissue architecture	Technically and analytically complex	Reconstructing the multilayer regulatory organization of inflammatory niches

IMC, imaging mass cytometry; DSP, digital spatial profiling.

### 2.5 Analytical workflow: from spatial measurements to inflammatory niches

Platform selection should be coupled to an analysis plan because the appropriate computational workflow depends on whether the data consist of multicellular capture spots or individually localized transcripts. For spot-based data, cell2location integrates single-cell references to estimate cell-type abundance within each location (33), whereas Baysor reconstructs cell boundaries and assigns transcripts in imaging-based datasets (34). Once cells or cellular compositions have been resolved, CellCharter can identify and compare multicellular niches (35), while Squidpy provides neighborhood graphs and spatial statistics that can be combined with distance-to-lesion measurements to analyze spatial gradients (36). When multiple tissue sections are integrated, PRECAST aligns spatially informed representations while accounting for inter-section batch effects (37); the sensitivity of downstream domains, gradients and communication networks to segmentation, deconvolution and batch correction should therefore be evaluated before biological interpretation.

## 3 Spatial multi-omics across brain diseases

### 3.1 Alzheimer’s disease and tauopathies - the amyloid plaque inflammatory niche

In Alzheimer’s disease (AD), spatial omics has reframed reactive gliosis, cytokine elevation and complement activation as a structured, distance-dependent microenvironment organized around the amyloid plaque, widely termed the plaque niche. To our knowledge, one of the first spatial transcriptomic studies in AD was performed in App^NL-G-F^ knock-in mice, with key findings subsequently validated by *in situ* sequencing in mouse and human brain sections. This study identified a network of 57 plaque-induced genes (PIGs) that became progressively co-expressed within approximately 100-μm-diameter tissue domains centered on amyloid plaques and scaled with local Aβ burden (8). This program was multicellular, spanning microglia and astrocytes, and was enriched for complement, endosomal–lysosomal, oxidative-stress and inflammatory genes, with the complement cascade among the most dysregulated; an earlier oligodendrocyte and myelin co-expression module appeared at lower plaque burden, suggesting a temporally ordered tissue response (8). This program was restricted to plaque-adjacent tissue domains and was absent from control tissue (8).

Higher-resolution imaging-based methods subsequently sharpened this diffuse 100-μm domain into a layered architecture. STARmap PLUS, which co-maps approximately 2,766 transcripts with amyloid and phospho-tau protein in the same section at near-subcellular resolution, demonstrated that the plaque niche is organized as a core–shell structure in which disease-associated microglia (DAM) directly contact the Aβ core while disease-associated astrocyte-like cells and oligodendrocyte precursor cells occupy concentric outer shells around the plaque–DAM complex (32). Microglia showed the strongest enrichment within 20 μm of plaques, encompassing the 0–10-μm and 10–20-μm annuli, whereas astrocytes, oligodendrocytes and oligodendrocyte precursor cells were enriched predominantly at 10–30 μm, directly demonstrating cell-type-specific radial organization within the plaque niche (32). The same study placed tau pathology in a separate spatial compartment, with hyperphosphorylated tau accumulating mainly in CA1 excitatory neurons alongside infiltration of oligodendrocyte subtypes into hippocampal axon bundles, thereby visualizing the spatial divergence of the amyloid and tau responses within a single tissue (32). A recurring theme revealed by spatial analyses is that the transcriptional state of a glial cell is strongly associated with its position relative to pathology, providing information beyond cluster identity alone. Using Xenium with an immunometabolic gene panel across amyloid (5xFAD, APP/PS1) and tau (PS19) models and human Braak VI cortex, proximity to plaques was itself a strong predictor of state, with microglia closer to a plaque showing greater upregulation of glycolytic, lipid-catabolic and pro-inflammatory genes, indicating a plaque-localized immunometabolic reprogramming conserved between mouse and human (38). Distance-based analysis of human tissue similarly isolated a reactive SERPINA3^+^ astrocyte state whose marker intensity rose sharply within 50 μm of neuritic plaques and declined with distance, validated at the protein level, reframing the conventional homeostatic-versus-reactive dichotomy as the endpoints of a continuous spatial gradient (39).

Because spatial methods retain information on which cells are neighbors, they allow inflammatory cell–cell communication to be inferred under a proximity constraint rather than from dissociated mixtures. Analysis of the plaque niche supports a microglia-to-astrocyte signaling relationship in which complement components are partitioned across neighboring cell types, with microglia expressing C1qa, astrocytes Clu and oligodendrocytes C4, all localized to plaque-adjacent cells and confirmed by *in situ* hybridization (40). High-resolution work further showed that the astrocytic response was region-specific, with hippocampal plaque-associated astrocytes upregulating Itm2b, Cpe and C1qa in a pattern distinct from the cortical response, whereas the microglial DAM response was comparatively consistent across regions. The same study identified 130 candidate microglia–astrocyte ligand–receptor pairs, 11 of which overlapped with the original plaque-induced genes (40). These analyses convert complement from a bulk biomarker into a spatially organized, multicellular module operating within the niche. Antibody-based spatial proteomics has been essential for testing whether these mouse-derived concepts hold in the human brain, where microglial states differ from rodent models. Multiplexed ion beam imaging of human cortex defined a continuous microglial state continuum compartmentalized by local microenvironment, in which more active microglial states localized near amyloid plaques while the AD hippocampus globally skewed toward a less active, putatively dysfunctional state (41). Cyclic multiplexed immunofluorescence and imaging mass cytometry identified plaque- and tangle-associated glial states and flagged a CD163^+^ myeloid population near plaques as likely human-specific infiltrating cells rather than resident microglia, a distinction with no clear mouse counterpart (42, 43), while imaging mass cytometry also linked spatial associations between activated microglia and selectively vulnerable neuronal subtypes such as RORB^+^FOXP2^+^ neurons accumulating intraneuronal Aβ, connecting the inflammatory niche to regional neuronal loss (44).

Several lines of work have begun to move beyond correlation. Human plaque–glia niche mapping coupled to iPSC-derived microglia showed that Aβ-exposed microglia, but not astrocytes, recapitulated the spatially defined glial states *in vitro* and tied glia-high plaques to greater CD68 abundance and local synaptic loss (45), and functional studies grounded in the niche concept implicate C1q-dependent synapse elimination by both microglia and astrocytes around plaques, with astrocytic phagocytosis compensating when Trem2-dependent microglial clearance fails (46). Large human spatial and single-nucleus transcriptomic studies have extended these niche-level observations across genetic and sporadic AD cases (47, 48). More recently, integrated spatial transcriptomic, single-nucleus RNA-sequencing and Xenium analyses identified microglial transitions at the Aβ–tau inflection point that were associated with divergent trajectories toward dementia and cognitive resilience (49). Taken together, these studies define the plaque niche as a radially organized, complement-anchored and metabolically reprogrammed inflammatory microenvironment that is spatially separated from the neuron-centered tau response and partly divergent between mouse models and the human brain.

### 3.2 Multiple sclerosis — the chronic active lesion rim

In multiple sclerosis (MS), spatial omics has provided the clearest demonstration that neuroinflammation is not diffuse but compartmentalized into a defined anatomical structure: the chronically inflamed edge of the demyelinated white matter lesion. The conceptual groundwork was laid by MRI-informed single-nucleus RNA sequencing of lesion edges, which catalogued the glial and immune diversity at the leading edge of demyelination and defined two states central to progression, “microglia inflamed in MS” (MIMS) and “astrocytes inflamed in MS,” both carrying neurodegenerative transcriptional programs (9). A notable feature of that work was that the MIMS profile overlapped with reactive microglial states described in other neurodegenerative diseases, an early signal of the cross-disease convergence developed later in this review (9). Paramagnetic rim lesions (PRLs), defined by a peripheral paramagnetic rim on susceptibility-sensitive MRI, provide an *in vivo* imaging correlate of persistent lesion-edge inflammation and are associated with greater disability (9). However, PRLs should not be treated as synonymous with histopathologically defined chronic active lesions: PRLs are defined radiologically, whereas chronic active lesions are classified histopathologically by a demyelinated core surrounded by a rim of activated microglia and macrophages, The two categories are related but not identical (50). What single-nucleus data could not establish, however, was the spatial organization of these states relative to the lesion core and surrounding tissue, which is precisely the gap spatial methods closed.

Paired single-nucleus and spatial transcriptomic mapping of subcortical MS lesions then resolved the lesion into spatially distinct niches and showed that inflammation is progressively restricted to the rim as a lesion matures from active demyelination toward the chronic active stage (51). This work defined the chronic active lesion as a hypocellular, demyelinated core encircled by an inflamed rim populated by CD68- and CD163-positive myeloid cells containing myelin degradation products, with iron-laden microglia and macrophages accumulating specifically at that rim (51). Complementary lesion-edge profiling localized osteopontin (SPP1), ferritin light chain (FTL) and apolipoprotein C1 (APOC1) to microglia situated at the rim, while astrocyte-focused analysis of the lesion core identified a SPAG17+ ciliated astrocyte state of the glial scar, assigning different glial programs to anatomically separate lesion compartments (51). The most recent single-cell-resolution spatial atlas, generated by MERFISH on archived human lesions, further resolved two study-defined microglial states: a lipid-laden state termed MIMS2, marked by GPNMB, ABCA1, ABCG1 and PLIN2 and localized predominantly to the lesion rim, and a stress- and interferon-associated state termed MIMS3, marked by FTH1 and heat-shock and DNA-damage transcripts and enriched in the inactive demyelinated core, demonstrating that even within a single lesion, microglial identity is partitioned by position (52). The same study used spatially resolved mapping to delineate a discrete rim domain interposed between the demyelinated core and periplaque white matter, formalizing the rim as a measurable spatial entity rather than a histological impression (52).

Beyond cataloguing states, spatial profiling has reframed the rim as an actively maintained inflammatory niche sustained by spatially organized cellular interactions. Integrated single-nucleus and spatial analyses inferred cell–cell communication signatures distinguishing lesion from non-lesion tissue and traced the conversion of a homeostatic microenvironment into a pathogenic one along the lesion edge (51). The single-cell-resolution atlas extended this to tissue-resident lymphocytes, a sparse population previously difficult to characterize, and proposed that their interactions with rim microglia and astrocytes help sustain the compartmentalized inflammatory state, supporting a lymphocyte–microglia–astrocyte axis operating locally at the rim (9). Spatial mapping has also linked the rim niche to the failure of repair: lipid-laden, dysfunctional microglia arising from impaired efflux of myelin-derived cholesterol are positioned to perpetuate demyelination and obstruct remyelination, tying the spatial accumulation of foamy microglia at the rim to lesion non-resolution (52). These observations recast smoldering MS inflammation as a self-sustaining spatial circuit in which the physical co-localization of specific glial, immune and lymphocyte states maintains chronic activity.

Animal and longitudinal models have begun to convert these spatial associations into causal and dynamic mechanisms, addressing the central limitation that postmortem human tissue offers only static endpoints. Working from the spatially defined foamy-microglia state, conditional deletion of the cholesterol transporters Abca1 and Abcg1 in microglia during experimental autoimmune encephalomyelitis (EAE) increased the formation of lipid-storing phagocytes and worsened disease, whereas pharmacological enhancement of sterol efflux reduced foam-cell formation and inflammatory demyelination, establishing the rim-associated lipid phenotype as a driver rather than a bystander of persistent inflammation (52). Spatial transcriptomics of EAE spinal cord independently showed a stage-dependent shift from a widespread cytokine and chemokine response early in disease to focal glial accumulation within white matter lesions later, mirroring the human progression toward compartmentalized inflammation (53). Longitudinal, MRI-informed spatial profiling of MS-like lesions in the marmoset further captured perilesional microenvironments through formation and resolution, assigning central roles to lesion-associated astrocytes, oligodendrocyte precursor cells and ependyma, and providing the temporal dimension that human cross-sectional studies lack (54). Finally, spatial transcriptomics has connected the rim to a senescence program, revealing spatial zonation of senescence-like signatures across lesion cores, rims and periplaque regions and identifying microglia as especially vulnerable to inflammation-induced senescence, partially reversible with CNS-penetrant anti-inflammatory drugs (55). Taken together, the MS literature converts the lesion rim from a neuropathological landmark into a spatially defined, iron- and lipid-loaded, lymphocyte-supported inflammatory niche whose maintenance and resolution can now be studied, and perturbed, *in situ* — a structure that parallels the radial organization of the AD plaque niche while differing in its myelin-centered chemistry and its tight coupling to remyelination failure.

### 3.3 Stroke and acute injury — the ischemic core and penumbra

Ischemic stroke offers the most anatomically explicit example of spatially compartmentalized neuroinflammation, because the pathology itself is organized as concentric zones of decreasing injury - a necrotic core, a surrounding peri-infarct penumbra of reversibly damaged tissue, and adjacent normal-appearing parenchyma - that have long been defined clinically but only recently resolved at molecular and cellular detail. Spatial studies have resolved the established core–penumbra anatomy into zonally distinct inflammatory programs, linking glial and immune states to their location relative to the infarct (56). Spatial omics has supplied exactly this zonal resolution, and in doing so has reframed post-stroke inflammation as a spatially organized program in which a cell’s location relative to the infarct is strongly associated with whether it exhibits destructive or reparative features, beyond what can be inferred from lineage alone.

The defining demonstration came from paired single-cell and spatial transcriptomic profiling of the ischemic mouse brain, which partitioned microglia into two spatially and functionally opposite populations: ischemic core-associated microglia (ICAMs) within the necrotic tissue and ischemic penumbra-associated microglia (IPAMs) in the surrounding peri-infarct zone (10). ICAMs, driven by damage-associated molecular patterns and HMGB1 signaling, ran a glycolysis-fueled pro-inflammatory program and expressed high levels of cytokines and chemokines, whereas IPAMs displayed oxidative-phosphorylation signatures and protective, myelin-supporting features correlated with oligodendrocyte survival and remyelination (10). These juxtaposed microglial states define a position-associated destructive-to-reparative spectrum within ischemic tissue. Astrocytes showed a parallel spatial dichotomy: integrated Visium, single-cell and single-cell spatial profiling resolved proximal versus distal astrocyte populations relative to the infarct, with proximal astrocytes distinguished by altered lipid handling and enriched Apoe and Fabp5 expression, defining the molecular boundary of the forming glial scar in situ (57). High-plex Visium annotation further mapped the cellular composition of these zones, localizing microglia, macrophages and monocytes preferentially to the proximal peri-infarct region alongside infiltrating neutrophils, T cells and other peripheral populations, thereby placing the resident–peripheral immune interface at a defined spatial position (58).

Because spatial data retain neighbor relationships, stroke studies have used them to convert inflammatory cell–cell communication from a list of expressed ligand–receptor pairs into spatially anchored signaling within the peri-infarct niche, often with a reparative emphasis that distinguishes stroke from the chronically destructive AD and MS niches. Spatially informed CellChat analysis identified a galectin-9 (Lgals9)-Cd44 axis in which microglia and macrophages signal to oligodendrocyte precursor cells to promote their differentiation and remyelination, with disruption of this axis impairing repair, assigning a pro-regenerative function to a specific intercellular interaction localized to the peri-infarct area (10, 58). Spatial proteomics has reinforced the zonal model at the protein level: high-plex digital spatial profiling of the ischemic core border, peri-infarct and peri-infarct normal tissue revealed distinct, spatially co-localized protein signatures, including loss of neuronal MAP2 and NeuN and gains in autophagic and immune-invasion markers specifically at the core border, demonstrating that this functional zonation is encoded in protein as well as transcript (59). Together these analyses portray the penumbra not as a passive transition zone but as an actively organized niche where the balance of pro-inflammatory and pro-reparative signaling is set by position.

The acute and experimentally tractable nature of stroke has allowed spatial findings to be linked to intervention more readily than in chronic human neurodegeneration. Spatial transcriptomic profiling of penumbral microglia, astrocytes and neurons was used to show that histone deacetylase inhibition remodels microglial dynamics and rescues survival-associated neuronal programs in the penumbra after middle cerebral artery occlusion, with co-localized immunofluorescence validation and behavioral improvement, tying a spatially defined penumbral state to a therapeutic effect (60). Reviews synthesizing this rapidly growing literature now frame the core-versus-penumbra microglial dichotomy and the galectin–CD44 repair axis as organizing principles for stroke neuroimmunology and as candidate targets for promoting penumbral salvage (56). The central limitation remains that most datasets derive from rodent occlusion models profiled at single time points, so the temporal trajectory from acute inflammation to scar maturation and repair, and its conservation in human tissue, are still incompletely mapped. Even so, the stroke literature establishes the ischemic core–penumbra structure as a spatially compartmentalized inflammatory niche in which the same cell types adopt opposing destructive or reparative identities according to their distance from the infarct - a zonal organization that complements the radial plaque niche of AD.

### 3.4 Other diseases - glioma, aging, and emerging evidence in infection and autoimmunity

The plaque, rim and infarct niches discussed above are the best-characterized examples, but spatial omics has begun to extend the niche concept to a wider set of conditions in which neuroinflammation is organized in space, albeit on a thinner and faster-moving evidence base. These emerging cases test the broader applicability of the spatial niche framework beyond the three best-characterized diseases.

Glioma, and glioblastoma in particular, provides the most developed of these additional examples and connects neuroinflammation explicitly to the tumor immune microenvironment (61). Paired single-nucleus and Visium profiling of human glioblastoma resolved the tumor into spatially segregated niches and showed that the perinecrotic niche is markedly immunosuppressive, enriched for IL-10 and granzyme-A signaling, whereas the perivascular niche is comparatively pro-inflammatory, establishing opposing immune polarities at defined anatomical positions within a single tumor (62). Hypoxia emerged across studies as a principal organizer, producing a concentric arrangement of malignant and immune cell states radiating from necrotic cores toward vasculature (11). Spatially resolved myeloid mapping further linked the ontogeny and topology of tumor-associated macrophages to outcome, with microglia-like macrophages accumulating at infiltrative margins and monocyte-derived macrophages dominating hypoxic cores, and identified survival-associated myeloid neighborhoods and variable lymphoid access at tumor borders (11). These analyses recast glioma immune evasion as a property of niche-specific adjacency between myeloid states and malignant programs rather than of bulk immune composition.

Autoimmune and infectious encephalitides remain the least spatially characterized category, and most current evidence is indirect or derived from models rather than from large human spatial cohorts. The clearest spatial data come from infection-linked aging studies: spatial transcriptomics of mouse and human brain following influenza A pneumonia identified age-related neuroinflammation concentrated in white matter, involving microglia and CD8^+^ T cells, and showed that peripheral viral infection can accelerate the expansion of microglia expressing a DAM-like transcriptional program within the CNS microenvironment (63). This work is notable for connecting a peripheral immune challenge to a spatially localized central inflammatory response, a theme directly relevant to the perivascular and border niches treated elsewhere in this review. Dedicated single-cell-resolution spatial mapping of classical autoimmune encephalitis or of perivascular antigen-presenting niches in human tissue is still largely lacking, and represents one of the clearer gaps where the methods established in MS and glioma could be productively applied.

**Figure 1:**
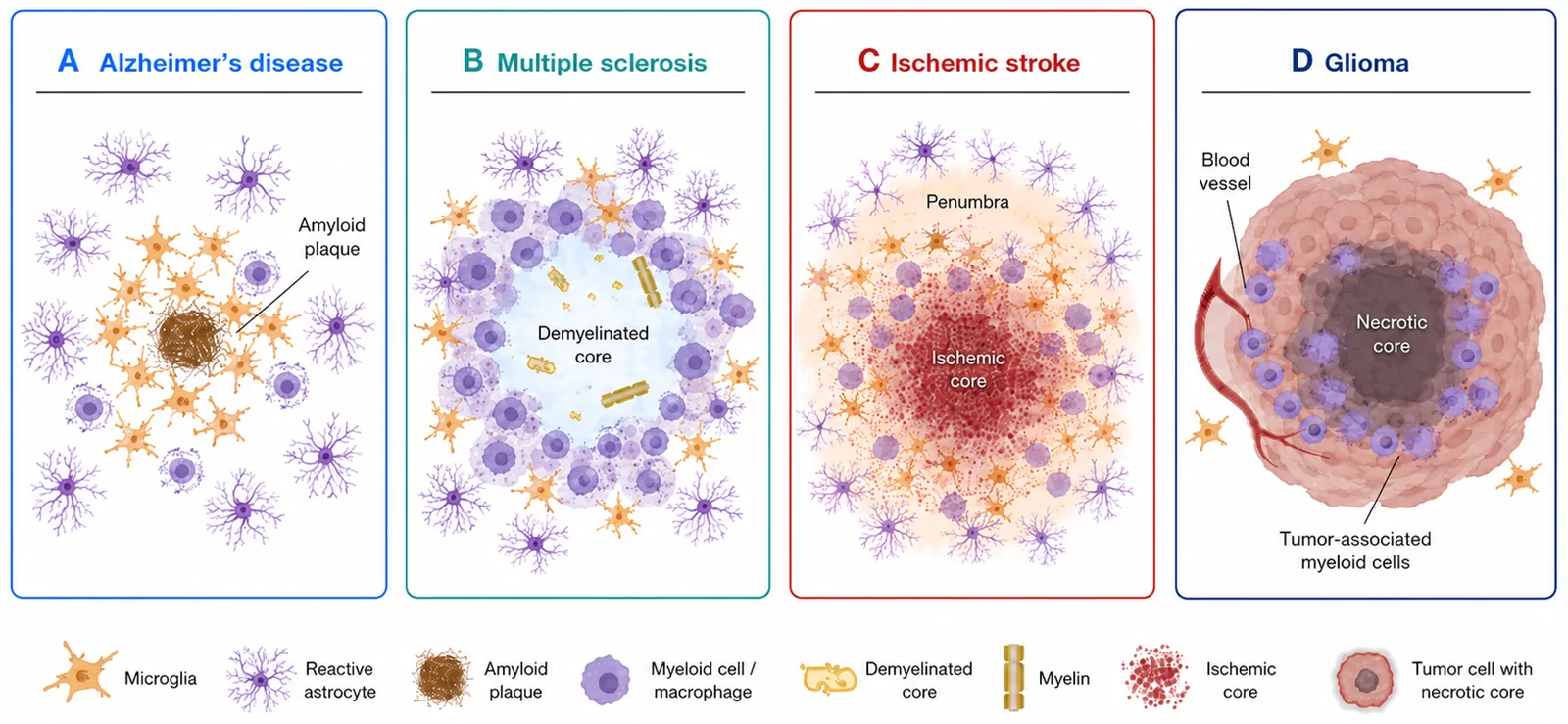
Spatially structured inflammatory heterogeneity across major brain diseases. Alzheimer’s disease is represented by an amyloid plaque-centered inflammatory niche surrounded by microglia and reactive astrocytes. Multiple sclerosis is characterized by a demyelinated core and an inflamed lesion rim enriched with myeloid cells and reactive glia. Ischemic stroke shows core–penumbra inflammatory zonation, with spatially distinct destructive and reparative cellular programs. Glioma contains necrotic and perivascular tumor niches shaped by tumor-associated myeloid cells and reactive glial components. Together, these examples illustrate that neuroinflammation is organized around local pathological structures rather than distributed uniformly across tissue.

The aging brain itself, finally, can be read as a chronic, low-grade analogue of the focal niches above, with white matter as its principal site. Spatial transcriptomics of young, middle-aged and old mouse brain found that age-associated inflammatory upregulation and synaptic downregulation were most pronounced in white matter fiber tracts, with signatures of microglial activation, astrogliosis, complement activation and myeloid infiltration, and confirmed physical interaction of activated microglia with fiber tracts alongside myelin loss (64). Human studies converge on the same compartment: deep subcortical and periventricular white matter lesions, an independent dementia risk factor, are marked by CD68^+^ microgliosis and dysregulated immune-response programs, and perivascular microglia accumulate in white matter in vascular and post-stroke dementia (65). Together these observations frame aging-associated neuroinflammation as a spatially biased process favoring perivascular white matter, providing the chronic substrate on which the more sharply demarcated disease niches form, and reinforcing that spatial position, here at the scale of tract and vessel rather than lesion, again governs where inflammation concentrates (**Figure 1**, **Table 2**).

**Table 2:** Representative spatial inflammatory niches across brain diseases.

Disease context	Species/tissue source	Principal spatial modality	Spatial niche and dominant features	Key spatial insight	Orthogonal/functional validation
Alzheimer’s disease (8)	AD-model mouse brain; mouse and human brain sections used for confirmation	Capture-based spatial transcriptomics and *in situ* sequencing	Amyloid plaque-associated niche containing plaque-proximal microglia, reactive astrocytes, and oligodendroglial responses	Glial states are spatially organized around amyloid plaques	Orthogonal: yes—*in situ* sequencing in mouse and human sections; Functional: no
Multiple sclerosis (9, 51, 52)	Human postmortem subcortical white-matter lesions; EAE mice used for functional testing	Spatial transcriptomics integrated with snRNA-seq; MERFISH	Demyelinated core, inflammatory rim enriched in lipid-laden MIMS2 microglia, stress-associated MIMS3 microglia in the inactive core, reactive astrocytes, and local immune interactions	Distinct glial and immune states are compartmentalized across the lesion core, rim, and periplaque white matter	Orthogonal: yes—histology and immunostaining; Functional: yes—microglial *Abca1/Abcg1* deletion in EAE
Ischemic stroke (10, 58)	Mouse ischemic brain	Spatial transcriptomics integrated with single-cell transcriptomics	Ischemic core–penumbra–peri-infarct zonation containing spatially distinct microglia, astrocytes, and infiltrating immune cells	Destructive and reparative inflammatory programs occupy distinct core, penumbra, and peri-infarct compartments	Orthogonal: yes—histological and protein-level validation; Functional: yes—intervention targeting the Lgals9–Cd44 axis
Glioma (11, 62)	Surgically resected human glioblastoma tissue	Spatial transcriptomics, spatial proteomics, and paired snRNA-seq	Perinecrotic, hypoxic, and perivascular niches containing distinct malignant and tumor-associated myeloid-cell states	Local tumor compartments are associated with distinct immune and malignant-cell programs	Orthogonal: yes—histopathology and spatial proteomics; Functional: no direct perturbation
Aging and white-matter injury (64)	Young, middle-aged, and old mouse coronal brain sections from both sexes	10x Visium spatial transcriptomics	White-matter tract and perivascular inflammatory niches containing activated microglia, reactive astrocytes, and myelin-associated injury	Aging-related inflammatory programs are preferentially enriched in white-matter fiber tracts	Orthogonal: yes—immunofluorescence, confocal imaging, flow cytometry, and ex vivo dMRI; Functional: no

AD, Alzheimer’s disease; dMRI, diffusion magnetic resonance imaging; EAE, experimental autoimmune encephalomyelitis; MERFISH, multiplexed error-robust fluorescence *in situ* hybridization; snRNA-seq, single-nucleus RNA sequencing.

## 4 Emerging principles

### 4.1 Spatial location refines disease-defined cell identity

A consistent message across the disease-specific niches reviewed above is that the inflammatory phenotype of a glial cell is strongly associated with its position relative to pathology. In several datasets, spatial position predicts cell state beyond disease category or transcriptomic cluster assignment. This relationship has a firm prior in basic microglial biology: the mature microglial signature is not entirely cell-intrinsic but is shaped by local cues, such that microglia rapidly lose characteristic features when removed from the brain and partially regain them following re-engraftment into intact parenchyma, while peripherally derived macrophages can acquire microglia-like features within the CNS environment (3, 66, 67). Spatial omics extends this concept from the homeostatic brain to inflamed tissue by revealing close associations between local microenvironment, distance from pathology and inflammatory state at micrometer-scale resolution. These observations are consistent with, but do not by themselves establish, position-dependent regulation of glial phenotypes.

The most direct evidence is that physical distance to a pathological focus predicts transcriptional state in a graded, quantitative manner. In the amyloid plaque niche, proximity to a plaque is itself a strong predictor of microglial state, with cells nearer the plaque progressively upregulating glycolytic, lipid-catabolic and pro-inflammatory programs (38), while a reactive astrocyte state defined by SERPINA3 expression shows marker intensity that rises within tens of micrometers of a plaque and declines with distance (39). The same logic structures pathology that is not centered on a point source: in the multiple sclerosis lesion, distinct microglial states are partitioned between the inflamed rim and the demyelinated core, and spatial gradient mapping resolves a discrete rim domain interposed between core and periplaque tissue (51, 52). In ischemic stroke the principle is most starkly illustrated, because two states of the same cell type—ischemic core-associated and penumbra-associated microglia-occupy adjacent zones separated by a few hundred micrometers yet run opposite, destructive versus protective, metabolic and inflammatory programs, with a parallel proximal-versus-distal split among astrocytes distinguished by lipid handling and *Apoe*/*Fabp5* expression (10, 57). Across these settings, position relative to injury is strongly associated with where a cell falls along the destructive-to-reparative spectrum, although spatial association alone cannot determine whether location causes the observed state.

This recurrent association between spatial position and transcriptional state refines the way inflammatory cell states should be classified. Dissociation-based single-cell studies have catalogued numerous discrete microglial and astrocytic states—including disease-associated microglia, microglia inflamed in MS and reactive astrocyte subtypes—but assign identity by transcriptomic clustering after spatial information has been lost, while microglia can begin remodeling their transcriptome within hours of isolation (3, 66). Spatial data suggest that some nominally distinct states may be better described as positions along continuous environmental gradients rather than as fixed, mutually exclusive cell types: membership in a “disease-associated” or “inflamed” category frequently tracks with location within the niche, and high-resolution profiling repeatedly identifies proximity to pathology as a predictor of state (38, 52). This does not diminish the value of cell-state catalogues but indicates that spatial context should be considered alongside intrinsic cellular identity. It also raises the testable hypothesis that altering local signals may shift cells between beneficial and harmful states, a possibility that requires direct perturbational validation.

The convergence of this principle across etiologically unrelated diseases is itself significant. A point-source amyloid deposit, an autoimmune demyelinated edge and an ischemic boundary are each associated with graded, position-dependent inflammatory states in resident glia, indicating that the relationship between spatial position and inflammatory phenotype is a recurrent organizing feature of CNS inflammation rather than a disease-specific observation. This convergence also has an important methodological implication: because glial inflammatory phenotypes are continuously shaped by local tissue cues and can be rapidly distorted by dissociation, *in situ* measurement is not merely a higher-resolution complement to single-cell sequencing, but a biologically more faithful approach for defining inflammatory states within their native microenvironment (68).

### 4.2 Neuroinflammation exhibits spatial zonation and compartmentalization

If cellular inflammatory state is strongly associated with spatial location, its corresponding tissue-scale organization can be understood in terms of spatial zonation and compartmentalization. Spatial omics reveals that inflammatory programs are differentially distributed across lesion cores, rims, perilesional tissue, and other anatomically defined microenvironments, extending beyond a simple binary division between inflamed and uninflamed regions. The conventional vocabulary of neuropathology-lesion versus normal tissue, active versus inactive, reactive versus resting-imposes categorical boundaries that bulk and histological methods inherited and reinforced. Spatial omics consistently reveals instead that inflammatory programs vary smoothly with distance from a pathological focus, that the boundaries between “lesion” and “normal” tissue are gradients rather than edges, and that biologically meaningful inflammatory activity extends well into tissue that appears normal by conventional staining.

The clearest demonstrations come from explicitly distance-based analytic strategies that treat proximity to pathology as a continuous variable. In human Alzheimer’s cortex, calculating each transcriptomic spot’s distance to the nearest neuritic plaque and testing expression as a function of that distance-rather than dichotomizing tissue into plaque and non-plaque-identified genes such as SERPINA3 and metallothioneins whose expression declines continuously from the plaque outward, with the reactive astrocyte signature highest within roughly 50-150 μm and falling off thereafter (39). The same continuous logic is captured by gradient-mapping algorithms applied to demyelinating lesions: in chronic active multiple sclerosis lesions, computational gradient mapping of glial states delineates a rim domain that lies on a continuum between the demyelinated core and the periplaque white matter, with pathway activity shifting progressively from oxidative-stress and neurodegeneration programs in the core, through inflammatory, lipid-metabolic and interferon signaling at the rim, to immune-regulatory and stress-response programs in the surrounding white matter (52). These findings demonstrate spatial zonation, with distinct molecular programs distributed across the demyelinated core, inflammatory rim, and periplaque white matter.

A particularly consequential feature of these gradients is that inflammation routinely penetrates beyond the visible margin of pathology into so-called normal-appearing tissue, a phenomenon that categorical lesion definitions are structurally unable to capture. Spatial transcriptomics of meningeal inflammation in an autoimmune model showed that inflammatory gene signatures originating in the meninges extend with variable penetrance into the adjacent brain parenchyma, providing a spatial substrate for the subpial gradient of cortical injury long observed in multiple sclerosis (69). Within white matter lesions, subtle expression shifts and communication gradients in periplaque white matter parallel parenchymal inflammatory changes seen in experimental models, indicating that the periplaque region is not a passive boundary but the leading slope of an inflammatory gradient (70). The recurrent finding that “normal-appearing” tissue carries graded inflammatory signal reframes it as the low end of a continuum rather than as a true unaffected control, with direct implications for how lesion burden and disease activity should be quantified.

Spatial compartmentalization is particularly evident when opposing functional programs occupy adjacent anatomical zones. In ischemic stroke, the necrotic core, penumbra, and peri-infarct tissue contain distinct microglial metabolic and functional programs: glycolysis-associated, pro-inflammatory, and destructive states predominate in the core, whereas more oxidative, myelin-supporting, and reparative states are enriched in the penumbra (10). Together with the plaque- and lesion-associated organization described above, these findings show that inflammatory states are structured according to their anatomical relationship with local pathology. Spatial position therefore provides an informative context for interpreting inflammatory state, although it should not be considered causally determinative.

### 4.3 Spatial proximity refines our view of cell–cell communication

The gradients described above may reflect, and potentially be maintained in part by, signaling between neighboring cells, making cell–cell communication an important candidate mechanism within inflammatory niches. Yet inference of such communication has been one of the least reliable products of dissociation-based transcriptomics, and this is precisely the inferential gap that spatial data are best positioned to close. Conventional ligand–receptor methods built for single-cell data, including the original implementations of widely used tools, score communication from the coordinated expression of a ligand in one population and its receptor in another, while ignoring whether those populations are anywhere near each other in tissue (71, 72). Because physical contact or short-range diffusion is required for most paracrine and juxtacrine signaling, expression-only approaches may nominate interactions between cells that are not located within a biologically plausible signaling range. A recent preprint benchmark reported that expression-based and some spatially naïve methods retained substantial numbers of predicted interactions in randomized control data, whereas explicitly incorporating spatial proximity markedly reduced such predictions (73). These preliminary findings require further validation across datasets and methods, and spatial proximity increases the plausibility of inferred communication without establishing functional signaling.

Spatial transcriptomics supplies exactly the missing coordinate, and a substantial methodological literature has emerged to exploit it. Updated versions of standard tools now incorporate distance, and dedicated frameworks model communication as a function of spatial separation in increasingly explicit ways-weighting ligand–receptor signaling by distance, modeling ligand diffusion and competitive receptor binding, applying optimal transport to balance competing ligand and receptor species across space, or using knowledge-graph and neighbor-network approaches to score signaling only between spatially proximal cells (74–76). The shared principle across these methods is to restrict candidate interactions to cell pairs whose physical separation is compatible with the biochemistry of the signal, converting a list of co-expressed ligand–receptor pairs into a spatially plausible communication network. For neuroinflammation specifically, this matters because the inflammatory niche is defined precisely by which cells are co-localized, so that imposing the proximity constraint is equivalent to testing communication within, rather than across, the niche.

Applied to the disease niches reviewed in Section 3, spatially constrained inference has converted previously speculative signaling relationships into spatially anchored axes operating locally within the niche. In the Alzheimer’s plaque niche, proximity-aware analysis supports a microglia-to-astrocyte signaling relationship in which complement components are partitioned across neighboring cell types and nominates on the order of 130 candidate microglia–astrocyte ligand–receptor pairs enriched specifically in plaque-adjacent cells, a subset overlapping the plaque-induced gene network (8, 40). In ischemic stroke, spatially informed analysis localized a galectin-9–CD44 axis through which peri-infarct microglia and macrophages signal to oligodendrocyte precursor cells to promote differentiation and remyelination, assigning a reparative function to an interaction defined by its position in the penumbra (58). In multiple sclerosis, spatial profiling of the chronically inflamed rim supports a lymphocyte–microglia–astrocyte axis in which the physical co-localization of sparse tissue-resident lymphocytes with rim glia is proposed to sustain the compartmentalized inflammatory state (9, 52). In each case, spatial adjacency increases the biological plausibility that the nominated cell populations interact locally and potentially contribute to inflammatory progression or resolution.

Two caveats keep this advance honest and connect it to later sections. First, spatial proximity raises the plausibility of a communication event but does not prove it; co-localization and ligand–receptor co-expression remain correlative, and demonstrating that a nominated axis actually carries signal with functional consequence still requires perturbation, an argument developed in Section 5. Second, the proximity constraint is only as good as the underlying spatial resolution: at spot-level resolution a single capture area may contain several cells, so apparent “adjacency” can itself be an artifact of resolution, and transcript diffusion can place signal where no cell sits-issues taken up among the analytical pitfalls in Section 5. With these limits acknowledged, the central point stands: communication in the inflammatory niche is interpretable only in light of where cells are, and the recurrence of distinct but analogously organized signaling axes—complement-anchored glial crosstalk in AD, a galectin–CD44 repair axis in stroke, a lymphocyte–glia axis in MS—reinforces the theme that etiologically different diseases construct their inflammatory microenvironments from a common spatial logic.

### 4.4 Shared spatial organization and disease-specific inflammatory architectures

Three principles emerge from the disease niches surveyed above: spatial position strongly predicts and is associated with inflammatory cell state, inflammatory activity often follows continuous gradients rather than exclusively discrete boundaries, and candidate cell–cell communication becomes more biologically plausible when evaluated under a spatial-proximity constraint. Together, these observations suggest a recurrent spatial architecture of neuroinflammation across several diseases with fundamentally different initiating factors. A misfolded protein deposit in Alzheimer’s disease, an autoimmune attack on myelin in multiple sclerosis, an acute loss of blood supply in stroke and a malignant clone in glioma are each associated with inflammatory microenvironments displaying related spatial features: a focal pathological region, enrichment of reactive glia in its immediate vicinity and progressive changes in inflammatory activity with distance. These recurring arrangements include the plaque-centered organization of Alzheimer’s disease, the core–rim–periplaque structure of chronic active multiple sclerosis lesions, the core–penumbra–peri-infarct gradient of ischemic injury and the perinecrotic-to-perivascular organization of glioma. Although these similarities do not demonstrate a common causal mechanism, they are consistent with a substantial contribution of the local microenvironment to the organization of inflammatory responses.

The depth of evidence supporting this convergence remains uneven across diseases. Position-associated cell states and spatial gradients have been reproduced most consistently in Alzheimer’s disease (8, 32, 38), multiple sclerosis (51, 52) and experimental stroke (10, 58), although the stroke evidence remains predominantly derived from animal models. Human tissue studies provide support for related spatial organization in Alzheimer’s disease (39, 41, 42), multiple sclerosis (51, 52) and glioma (11, 62), whereas evidence in infection-related neuroinflammation and aging remains more preliminary (63, 64). Moreover, proximity-defined communication remains largely inferential, and the functional consequences of spatial organization have been tested in only selected settings (46, 52, 60, 77). The proposed convergent architecture should therefore be regarded as an evidence-supported working model rather than as a universally established rule.

This spatial convergence is reinforced by independent molecular evidence that the cell states populating these niches are themselves shared across diseases. The disease-associated microglia signature was originally defined not in one disease but simultaneously across mouse models of Alzheimer’s disease, amyotrophic lateral sclerosis and multiple sclerosis, and its induction depends on the same TREM2–APOE axis in each (78, 79). This cross-disease conservation has since been demonstrated directly in human tissue: an integrated atlas of more than ninety thousand human brain immune cells spanning six neurological conditions identified disease-associated microglia and inflammatory-macrophage subsets shared across all pathologies, including an expansion of GPNMB-high microglia common to both multiple sclerosis and Alzheimer’s disease (80), while a separate resource profiling more than two hundred thousand human microglia from seventy-four donors across diverse neurological diseases found recurrent subsets enriched for the disease-associated microglial signature and validated them *in situ* by RNAscope and high-dimensional MERFISH (81). The lipid- and phagocytosis-centred character of this shared program is consistent across conditions and overlaps the lipid-laden myeloid states independently identified at the multiple sclerosis rim and the amyloid plaque (82). These molecular and spatial similarities suggest that related myeloid programs may occupy analogous positions within different inflammatory niches and potentially perform overlapping functions, although their functional equivalence remains to be established experimentally.

Framing neuroinflammation as a convergent spatial architecture carries consequences that distinguish this review’s stance from a disease-by-disease catalogue. Mechanistically, it suggests that the rules governing niche assembly—what recruits myeloid cells to a focus, what sets the astrocyte belt at its characteristic distance, what sustains complement signaling locally—may be partly disease-agnostic and therefore studiable as general principles rather than rediscovered separately in each pathology. Therapeutically, it raises the possibility that interventions targeting a shared niche feature, such as the lipid-metabolic state of lesion-central myeloid cells or a conserved glial communication axis, could generalize across diseases that are clinically unrelated, although whether the shared program is protective or harmful appears to be context-dependent and remains unresolved (82). The convergence must not be overstated: real differences remain in chemistry and kinetics—the myelin-centered lipid load of the multiple sclerosis rim, the acute reparative bias of the stroke penumbra, the immunosuppressive perinecrotic niche of glioma—and these distinctions matter for treatment. These differences are nevertheless compatible with a partially shared spatial theme whose generality remains to be established. Spatial omics provides a framework for testing this possibility by preserving the relationship between cellular state and tissue position. Moving from this still largely descriptive architecture to demonstrated causal mechanism remains the central unresolved challenge, taken up next.

## 5 From spatial maps to mechanisms

### 5.1 Spatial omics generates hypotheses, not causal proof

The preceding analysis establishes a powerful descriptive framework, but it is essential to be explicit about what that framework does and does not demonstrate. Every observation considered so far that a microglial state intensifies with proximity to a plaque, that inflammatory programs form gradients across a lesion, that two cell types expressing a complementary ligand and receptor lie adjacent within a niche—is fundamentally a statement of spatial association. Association is not causation, and the inferential distance between the two is the principal limitation of the spatial-omics enterprise as applied to neuroinflammation. A myeloid population enriched at a lesion core may drive the inflammation that defines that core, may be recruited as a downstream consequence of damage, or may even be attempting to contain the injury; the spatial map alone cannot distinguish these possibilities. The same ambiguity attends every gradient and every co-localized signaling axis: spatial data establish where inflammation is organized and which cells are positioned to interact, but not which arrangements are causes, which are consequences, and which are incidental.

This limitation is compounded by technical features of the methods that can manufacture the appearance of mechanism where none exists. Inference of cell–cell communication illustrates the problem directly: tools that score signaling from the mere co-expression of a ligand and its receptor, without spatial information, disregard the fact that interactions occur only between neighboring and not distant cells and consequently generate large numbers of false-positive predictions (5, 83). Spatial data are widely expected to constrain this problem, yet they introduce confounders of their own. A central one is limited resolution: many widely used platforms capture transcripts from areas containing several cells rather than from single cells—the 10x Visium spot, for example, spans roughly 55 μm-so that an apparent co-expression within one “spot” can reflect an admixture of distinct cells, and recovering single-cell composition requires deconvolution against external references that is itself imperfect (84). Even at subcellular resolution, segmentation errors routinely misassign transcripts between adjacent cells, and such mis-segmentation has been shown to confound differential expression, neighborhood analysis and ligand–receptor inference, often dominating the top predicted interactions with artifacts (85); systematic benchmarking across subcellular platforms similarly reports incomplete coverage and false positives as inherent limitations (86). Because postmortem human tissue and single-timepoint animal samples capture only a static snapshot, they additionally cannot establish the temporal ordering on which causal claims depend—whether a glial state precedes or follows the pathology it surrounds. None of this diminishes the value of spatial profiling; rather, it defines its proper role. The unique contribution of spatial omics is to generate spatially precise, mechanistically specific and therefore testable hypotheses—candidate driver cells, candidate signaling axes, candidate gradient-defining genes that would be invisible to dissociation-based methods. Converting those hypotheses into mechanism requires perturbation.

### 5.2 Validation strategies and a suggested workflow

The strongest spatial-omics studies already treat their maps as the starting point of a validation pipeline rather than as an endpoint, and these exemplars define what rigorous practice looks like. A first tier of validation confirms that the spatial relationship itself is real rather than a technical artifact, using orthogonal *in situ* methods—RNAscope, multiplexed immunofluorescence or spatial proteomics—to verify that the nominated cells genuinely co-localize and express the implicated molecules at the protein level. A second tier confirms cellular identity and origin, since deconvolution and clustering can misassign cells; single-cell sequencing and lineage tracing establish that the population in question exists and derives from the proposed source, a distinction that matters acutely where resident microglia must be separated from infiltrating monocyte-derived cells. The decisive tier is causal perturbation: conditional gene deletion, cell-type-specific manipulation, or pharmacological blockade of a candidate, followed by assessment of whether the inflammatory phenotype, pathology and behavioral outcome change as predicted.

Several studies have completed this full arc and serve as templates across different diseases. In multiple sclerosis, a lipid-laden, dysfunctional microglial state identified at the lesion rim was tested by conditionally deleting the cholesterol transporters Abca1 and Abcg1 in microglia during experimental autoimmune encephalomyelitis, which increased foamy-cell formation and worsened disease, while pharmacologically enhancing sterol efflux reduced foam-cell formation and inflammatory demyelination, converting a spatial correlation into a manipulable driver of persistent inflammation (52). In ischemic stroke, single-cell and spatial transcriptomics identified BST2-expressing astrocytes enriched specifically at the lesion border; astrocyte-specific Bst2 knockout then reduced astrocyte–microglia interactions, attenuated border formation and improved early neurological outcome, with the mechanism traced to BST2-driven C3 expression recruiting microglia through C3/C3aR signaling, and the authors proposing border-restricted BST2 as a more selective target than global complement inhibition (77). The same complement axis has been independently validated in stroke, where microglia-derived IL-1α, TNF and C1q drive neurotoxic astrocyte formation via C3/C3aR/NF-κB signaling and blocking this induction attenuates reactive gliosis and ameliorates functional deficits (87), and in infection-associated cognitive impairment, where astrocyte-specific C3 deletion and C3/C3aR blockade prevented microglial over-engulfment of synapses and rescued cognition (88).

The amyloid niche has been interrogated with comparable rigor. Fate mapping revealed robust plaque-associated type I interferon responses in microglia and other neural cell types. In the same primary study, microglia-specific Ifnar1 deletion attenuated postsynaptic terminal loss associated with selective engulfment, whereas neural Ifnar1 deletion restored presynaptic terminals and reduced plaque accumulation, providing cell-specific functional evidence that type I interferon signaling contributes to plaque-associated synaptic pathology (89). Single-cell spatial transcriptomics of the Trem2^R47H^ Alzheimer’s risk variant in the 5xFAD model further showed that this microglial mutation alters neuronal as well as glial transcriptomes specifically in relation to plaque proximity, linking a genetic perturbation to its spatial consequences in situ (90), while parallel work in human iPSC-derived microglia and chimeric mice established that TREM2 deletion abolishes the disease-associated microglial response and impairs plaque coverage and chemotaxis, grounding the spatially defined DAM state in a manipulable receptor pathway (91). Across these examples a common structure recurs: a specific, localized cellular or signaling hypothesis is generated by spatial profiling, and targeted *in vivo* perturbation tests it against pathology and behavior.

**Figure 2:**
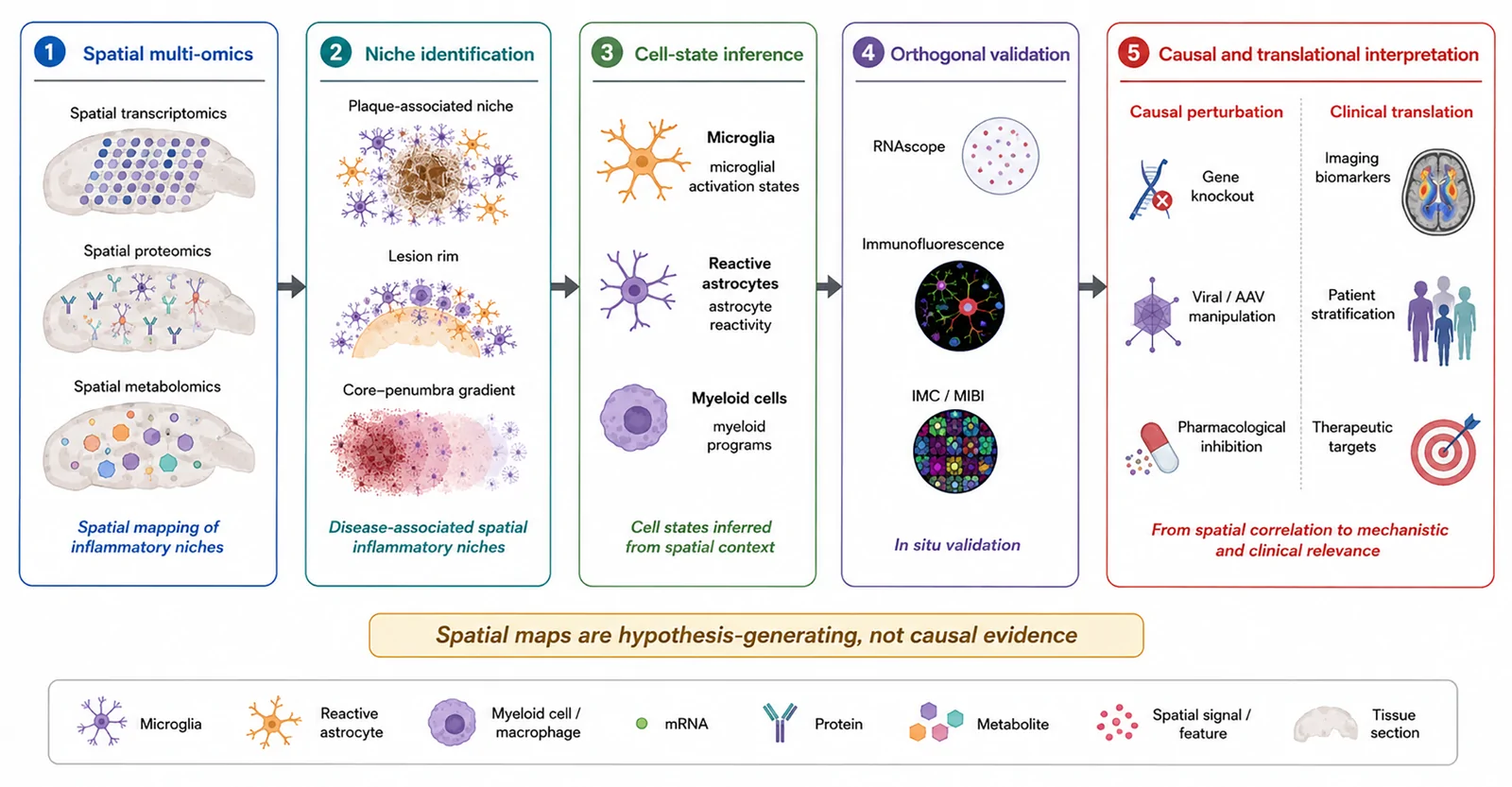
From spatial maps to mechanisms and translational interpretation in neuroinflammation. Spatial omics technologies identify inflammatory niches in intact brain tissue, including plaque-associated regions, demyelinated lesion rims and Ischemic core–penumbra zonation. These maps allow inference of spatially defined microglial, astrocytic and myeloid cell states. Because spatial association alone does not establish causality, candidate findings require orthogonal *in situ* validation and functional perturbation, including genetic, viral or pharmacological approaches. This workflow supports the transition from descriptive spatial maps to mechanistic interpretation, imaging biomarkers, patient stratification and therapeutic target discovery.

An emerging class of technologies promises to compress this workflow by bringing perturbation itself into spatial context. *In vivo* spatial CRISPR approaches now knock out neurodegeneration-risk genes within single cells of intact brain tissue while simultaneously reading out the perturbed cell’s transcriptome and the effects on its neighbors, distinguishing cell-autonomous from microenvironmental consequences without dissociating the tissue (92). By measuring perturbation effects *in situ*, these methods begin to close the gap between the spatial map and the causal test directly, rather than requiring a separate animal experiment for each hypothesis. A practical validation sequence therefore runs from spatial discovery of a candidate niche or axis, to single-cell resolution of its cellular composition and origin, to *in situ* confirmation of the spatial relationship by orthogonal staining, to ligand–receptor prioritization, to cell-type-specific perturbation, and finally to assessment of pathological, functional and behavioral consequences. Holding spatial findings to this standard is what separates a mechanistic account of neuroinflammation from an atlas of correlations, and it is the trajectory on which the field’s translational value ultimately depends (**Figure 2**).

## 6 Challenges and future directions

### 6.1 Key limitations: resolution, human tissue, functional validation

Three categories of limitation currently constrain how far the spatial picture of neuroinflammation can be pushed, and each follows directly from the analysis above. The first is technical resolution and the trade-offs that accompany it. No single platform yet delivers genome-wide coverage at true single-cell resolution across a large tissue area: sequencing-based methods capture the whole transcriptome but often pool several cells per spot, whereas imaging-based methods reach subcellular resolution at the cost of restricted gene panels, with cost and tissue coverage limiting throughput further (93, 94). Because the inflammatory niche is defined precisely by which cells are adjacent, these resolution limits and the segmentation and deconvolution errors that accompany them propagate directly into the biology, distorting estimates of cell composition, neighborhood structure and cell–cell communication (86). The neural context aggravates the problem: brain tissue is densely cellular and rich in interdigitated glial processes that are poorly captured by transcript-centred, soma-anchored measurements, so that the very astrocytic and microglial ramifications through which much inflammatory signaling occurs are systematically under-sampled.

The second limitation concerns human tissue itself. Much of the mechanistic confidence in this field still rests on animal models, yet the human-specific features documented by spatial proteomics—including myeloid populations and microglial states with no clear rodent counterpart—mean that model findings cannot be assumed to transfer. Human spatial studies face their own constraints: postmortem interval, fixation and agonal state degrade RNA and protein quality and vary across biobanks; cohorts are typically small and cross-sectional; and each specimen provides only a single static snapshot of what is a decades-long disease process, precluding the temporal ordering on which causal interpretation depends. The third limitation is the one emphasized throughout: the field remains weighted toward description, with the number of catalogued niches and states far exceeding the number that have been functionally validated. Although exemplary studies have closed the loop from spatial discovery to *in vivo* perturbation, these remain the exception, and the routine standard is still association rather than demonstrated mechanism. Progress in neuroinflammation will be measured less by additional maps than by the fraction of mapped features subjected to causal test.

### 6.2 Toward integrated, temporal, and clinically translatable spatial atlases

The most consequential near-term advance is the maturation of genuine spatial multi-omics, in which transcriptome, proteome, epigenome and metabolome are measured on the same tissue section rather than inferred across serial sections. For neuroinflammation this is not merely additive: co-measuring RNA with the protein aggregates, lipids and chromatin states that define a niche would directly correlate gene expression with pathological protein deposits within the same cellular neighborhood, allowing the regulatory logic of a niche to be read rather than assembled piecemeal (95, 96). Early frameworks already integrate RNA and protein on single tissue sections and place transcriptional, proteostatic, and epigenetic layers within common spatial coordinate systems, while analytic platforms now exist to process and jointly model such multimodal, multiresolution data. Studies outside the CNS have similarly integrated single-cell and spatial transcriptomic data to localize inflammation-associated stromal states within defined immune niches and relate these states to therapeutic response, illustrating the translational potential of multimodal spatial integration (97–101). Standardization is the precondition for this to become cumulative: spatial neuropathology still lacks the shared region-of-interest definitions, processing pipelines and reporting standards needed to compare datasets across laboratories, and explicit analytical guidelines—such as concentric plaque-centred sampling schemes that formalize the core-to-periphery gradient for multi-omic profiling—are beginning to address this gap (102).

Two further directions would convert static niche maps into dynamic, clinically meaningful models. The first is the addition of time and three-dimensional structure. Because human tissue offers only endpoints, longitudinal animal studies that profile the same lesion across formation and resolution are essential to establish causal ordering, as illustrated by serial, imaging-guided spatial mapping of demyelinating lesions through their life cycle; extending such designs and reconstructing niches in three dimensions would capture the inflammatory gradient as a process rather than a snapshot (54). The second is integration with the clinical layer. Linking spatial molecular features to *in vivo* imaging biomarkers—paramagnetic rim lesions being a prominent MRI-defined correlate of chronic rim inflammation—and to genetic and cognitive data would connect the microscopic architecture of the niche to measurable disease trajectories and treatment responses (50, 54, 103). Realizing this will depend heavily on computational and machine-learning methods able to handle the scale and dimensionality of spatial data, an area now developing rapidly across spatial biology (5, 104–107). The unifying goal is a shift from cataloguing where inflammation sits to predicting how a given niche will evolve and which of its features can be therapeutically redirected—the point at which the convergent spatial architecture described here would become a basis for intervention rather than description.

## 7 Conclusion

This review has traced how spatial omics is reshaping the study of neuroinflammation, from technologies that preserve tissue context to their application in defining the amyloid plaque niche in Alzheimer’s disease, the inflamed lesion rim in multiple sclerosis, the core–penumbra organization of ischemic stroke, and emerging inflammatory niches in glioma, infection and aging. Across these conditions, the shared principle is not a single conserved spatial blueprint, but the presence of spatially structured inflammatory heterogeneity associated with local pathology, cellular neighborhoods and tissue compartments. The resulting architectures and their functional implications remain disease-specific, ranging from relatively radial plaque-centered organization to rim-focused inflammation, functionally distinct ischemic zones and overlapping necrotic, hypoxic, invasive and perivascular tumor axes. Spatial position and proximity can therefore refine the interpretation of inflammatory cell states and prioritize biologically plausible interactions, but they do not establish functional equivalence or causality. Much of the current evidence remains descriptive, and the field has mapped far more niches than it has functionally tested. Its lasting value will depend on treating spatial maps as sources of testable hypotheses, validating them through cell-type-specific and *in situ* perturbation, and advancing toward same-section multi-omics, temporal profiling and integration with clinical trajectories. If the broader principle of spatially structured inflammatory heterogeneity is confirmed through direct comparative and functional studies, understanding how disease-specific niches are formed, maintained and resolved may reveal mechanistic and therapeutic opportunities across currently distinct neurological disorders.
